# Ferroelectric, Dielectric, Ferromagnetic, and Magnetoelectric Properties of BNF-NZF Bilayer Nanofilms Prepared via Sol-Gel Process

**DOI:** 10.1186/s11671-016-1592-5

**Published:** 2016-09-06

**Authors:** Kaixin Guo, Rongfen Zhang, Qingfeng Mou, Ruirui Cui, Chaoyong Deng

**Affiliations:** Key Laboratory of Electronic Composites of Guizhou Province, College of Big Data and Information Engineering, Guizhou University, Guiyang, 550025 China

**Keywords:** Sol-gel, Ferroelectric, Dielectric, Ferromagnetic, Magnetoelectric coupling, Deposition sequences

## Abstract

Bilayer magnetoelectric (ME) nanofilms composed of Bi_0.9_Nd_0.1_FeO_3_ (BNF) and Ni_0.55_Zn_0.45_Fe_2_O_4_ (NZF) were fabricated on the Pt(111)/Ti/SiO_2_/Si(100) substrates via sol-gel and a subsequent rapid thermal process with different growth sequences of BNF and NZF forming the following layered structures: BNF/NZF and NZF/BNF. The phase composition, microstructure, and ferroelectric, dielectric, ferromagnetic, and ME coupling properties of the composites were investigated at room temperature. Structural characterization by X-ray diffraction and scanning electron microscopy showed that there are no other impurity phases but BNF and NZF, and the nucleation barrier caused that it is easier for NZF and BNF to grow on each other rather than on the surface of Pt/Ti/SiO_2_/Si. The tests of the physical properties indicated that such heterostructures present both good ferroelectric, ferromagnetic, and dielectric properties and the in-plane ME coupling coefficient *α*_*E*_ at room temperature but some discrepancies also exist, which can be attributed to an interfacial effect, in other words, the deposition sequences of the constituent phases have a great influence on the properties of bilayer films.

## Background

Magnetoelectric (ME) multiferroic materials, which simultaneously exhibit ferroelectric, ferromagnetic, and ME coupling behaviors, have recently attracted extensive attentions for their significant potential applications in the next-generation novel multifunctional devices [1–3]. However, in comparison to bulk multiferroic composite [4], motivated by a pioneering work of Zheng et al. [5], most researches on ME materials have focused mainly on the nanostructured thin films which provide more degrees of freedom, such as lattice strain or interlayer interaction, to modify the ME behavior, and offer a way to investigate the physical mechanism of ME effect in nanoscale. There are two kinds of multiferroic connectivity structures, i.e., vertical heterostructures [6–8] (1-3-type structure) consisting of magnetic oxide (e.g., NiFe_2_O_4_, CoFe_2_O_4_) vertically embedded into a ferroelectric perovskite matrix (e.g., PbZr_1 − *x*_Ti_*x*_O_3_, BiFeO_3_, BaTiO_3_) and horizontal nanostructures [9, 10] (2-2-type structure) consisting of alternating layers of a ferroelectric perovskite and magnetic spinel. In comparison, the 2-2-type heterostructures are more simple and easier to take control of the growth of composite thin films but comparatively lower ME coupling coefficient *α*_*E*_ than the 1-3-type structures [11, 12], which can reduce effectively the leakage current density via isolating the magnetic layers with low resistance by insulating ferroelectric layers and the constraint strains suffered from the substrates could be released to some extent. Therefore, the 2-2-type laminar composite films have shown potential applications in applications of ME coupling.

Sol-gel technique, a most used and mature way to synthesize various function materials under the right conditions, has been widely used in scientific researches and technical exploitations and has a distinct advantage over other methods in the uniformity of film thickness, large-area fabrications, precise controls of chemical composition, etc. compared with pulsed laser deposition (PLD) [13, 14]. Bismuth ferrite doped with rare earth ions (Bi_1 − *x*_R_*x*_FeO_3_, *R* = rare earth ions), a representative single-phase multiferroic material at room temperature, which owns excellent ferroelectric, antiferromagnetic, and photovoltaic properties, has lately received widespread attentions since that it presents an extra-high ferroelectricity while epitaxially growing on single-crystal perovskite substrates [15]. Especially, Bi_0.9_Nd_0.1_FeO_3_ (BNF) shows better multiferroic and optical properties than pure bismuth ferrite (BFO), which has made BNF a suitable material for infrared detectors and optoelectronic devices [16]. Nickel zinc ferrite Ni_1 − *x*_Zn_*x*_Fe_2_O_4_ (NZF) with high-frequency, broadband, high-impedance and low-loss characteristics, has drawn much more attentions in recent years and has become the most widely used soft magnetic ferrite materials in the high-frequency range (1~100 MHz) [17]. Pt(111)/Ti/SiO_2_/Si(100) is comprised by a (100)-oriented Si (500 μm), SiO_2_ (500 nm), Ti (30 nm), and a (111)-Pt (100 μm); the (100)-Si exhibits a smaller crystalline interplanar spacing and greater surface density, and it can be easily cut experimentally. Experiments have shown that Pt nanolayer, prepared by magnetron sputtering, is usually (111)-preferred orientation and it presents a higher inoxidizability and makes the sols or precursors easier to spread over the whole surface of substrates.

Gu et al. have conjectured that magnetoelectric coupling exists in the BiFeO_3_-NiFe_2_O_4_ composite films in 2011 by the calculation for magnetic moment of NFO [18]. Therefore, in this work, we selected and synthesized heteroepitaxially multiferroic composite thin films of BNF-NZF (2-2-type structure) with different growth sequences on the Pt(111)/Ti/SiO_2_/Si(100) substrates by the sol-gel and rapid thermal process. Polarization and magnetization behaviors, dielectric properties, and the in-plane ME coupling characteristic at room temperature are studied in detail; meanwhile, the interfacial effect (the influence of layer deposition sequences on the properties of bilayer films) is also discussed, providing references for further study of material performance and device design.

## Methods

### Synthesis and Characterization

Using the sol-gel method, BNF with excess Bi-nitrate 15 mol % and NZF precursors were prepared mainly from the starting materials Bi(NO_3_)_3_·5H_2_O, Nd(NO_3_)_3_·6H_2_O, Ni(NO_3_)_2_·6H_2_O, Zn(NO_3_)_2_·6H_2_O, Fe(NO_3_)_3_·9H_2_O, and the solvent C_3_H_8_O_2_ (for the *Bi*-precursor, acetic anhydride [C_4_H_6_O_3_] is required to act as water removal agent). After aging, the precursor solution was passed through a syringe filter and spin-coated on the Pt(111)/Ti/SiO_2_/Si(100) substrates via a spinner operated at 4000 rpm for 30 s to form the first layer. These films were dried at 500 °C for 300 s to remove the residual organics, and then, they were annealed and crystallized at 650 °C for 420 s in rapid thermal process (RTP). The second layer was fabricated in the same way on the first layer, eventually forming anticipated 2-2-type lamellar films BNF/NZF (the first layer is BNF) and NZF/BNF (the first layer is NZF). In the end, Pt electrodes were deposited by a magnetron sputtering method on the surface of films using a metal mask with the diameter of 0.2 mm and heated at 500 °C for 300 s before measurements to improve the adhesion between the substrate and ferroelectric films.

**Fig. 1:**
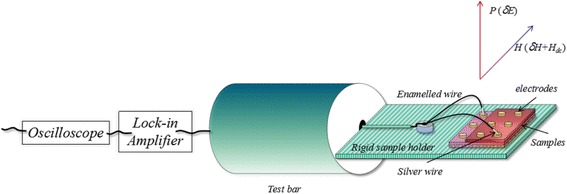
Schematic illustration of the experimental set-up for the in-plane ME measurement of the films

The crystalline phases and crystal structure of the films were determined by an X-ray diffractometer (XRD, model D/max-2500 V, Rigaku Co., Japan) with Cu Kα monochromatic radiation (*λ* = 0.154, 18 nm) at a scanning speed of 2(°)/min in steps of 0.02°. The microstructure of the films was obtained by a model S5500 scanning electron microscope (SEM) (Hitachi Co., Japan). The ferroelectric properties of the samples were analyzed by a model multiferroic 200-V Test System (Radiant Technologies), the ferromagnetism was obtained by a physical property measurement system (PPMS, Quantum Design), and the dielectric properties were measured by an impedance analyzer (Agilent 4294A) within the frequency range from 100 to 10^7^ Hz. The ME measurement of these films was performed in an open-circuit condition. The films were fixed in a rigid sample holder which was vertically suspended in air and placed between the poles of an electromagnet and a couple of *Helmholtz* coils, thus allowing application of both dc bias *H*_dc_ which could be changed in the range 0~5500 Oe and small superimposed ac magnetic fields *δH* of ±100 Oe under 20 kHz in parallel (see the experimental set-up shown in Fig. 1), as done in the measurements for bulk samples. The external measurement circuit was connected to the films through two silver wires bonded on the top and bottom electrodes, respectively. The two silver wires were very close to each other so as to minimize the loop of the set-up closed by the wires and samples. The magnetoelectric coupling coefficient *α*_*E*_ was calculated from the dielectric data using the relation [19]

1 $$ {\alpha}_E=\frac{\delta E}{\delta {H}_{\mathrm{ac}}}=\frac{\delta V}{t\cdot \delta {H}_{\mathrm{ac}}} $$

where *δV* is the induced ME voltage signals collected by a lock-in amplifier (SRS SR830), *t* is the thickness of films measured by a profilometer (BRUKER Dektak XT), and *δH*_ac_ is the alternating magnetic field signals collected by a Gauss/Tesla meter (REF F1218). All measurements were performed at room temperature.

## Results and Discussion

**Fig. 2:**
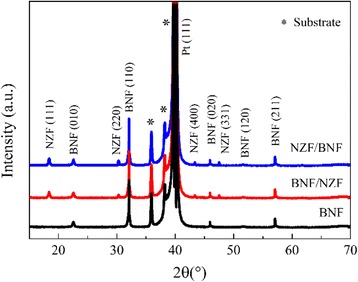
XRD patterns of the composite films

**Fig. 3:**
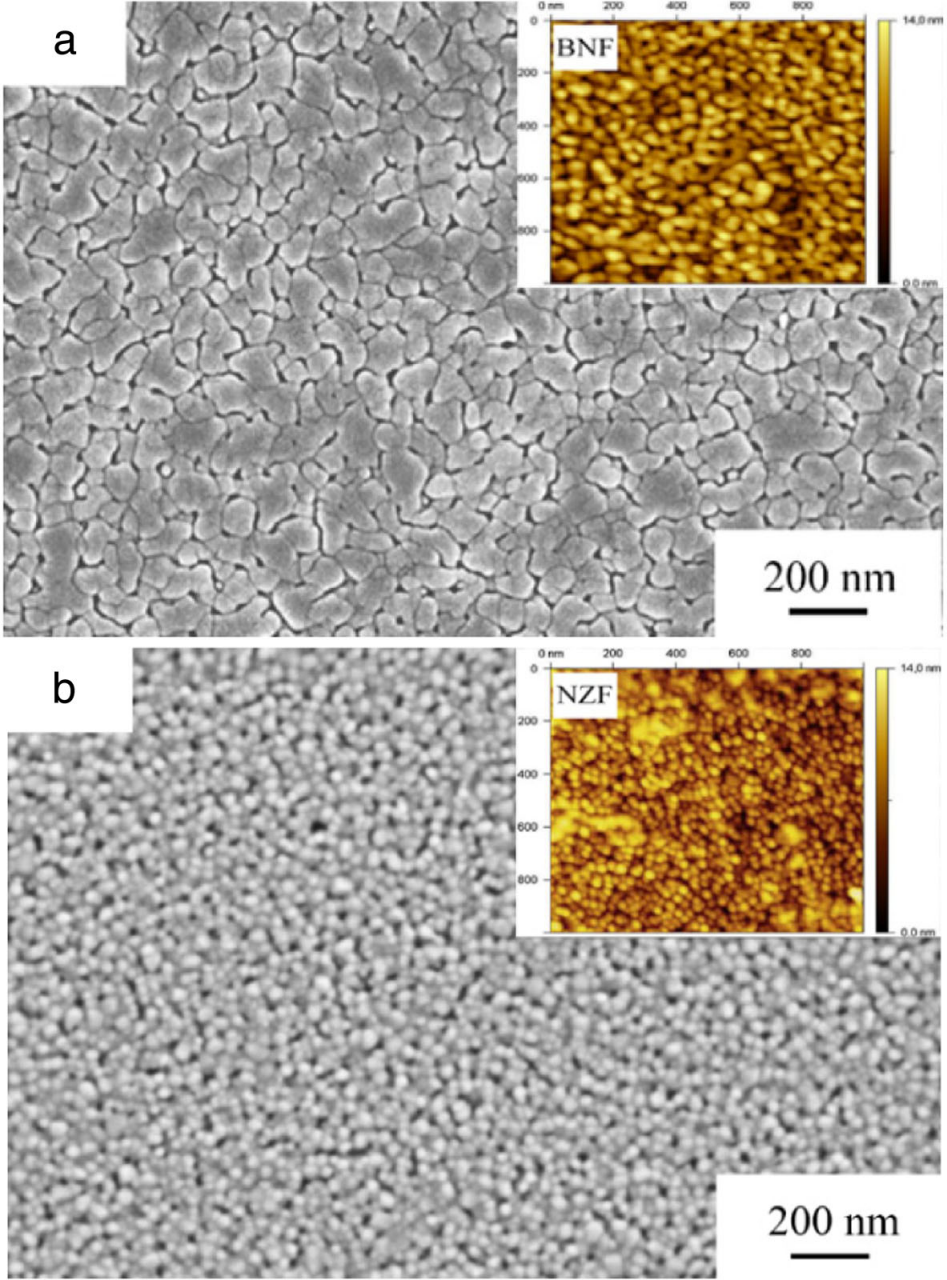
Microstructure of the top layer of the composite films: **a** BNF layer. **b** NZF layer. The *insets* are the AFM images of both the BNF layer and NZF layer

Figure 2 shows the X-ray diffraction (XRD) patterns of both BNF/NZF and NZF/BNF bilayer magnetoelectric composite films, containing an orthorhombic perovskite structure BNF with (110)-preferred orientations, a spinel structure NZF with (111) reflections, and no impurity or intermediate phases (such as Bi_2_Fe_4_O_3_ and γ-Fe_2_O_3_) apart from BNF and NZF. The full width at half maximum (FWHM) of the (110) peak is 0.264 in BNF/NZF but 0.265 in the NZF/BNF samples, and the (111) diffraction peak is all 0.252; then, the lattice parameters can be figured out, *a*_BNF_ = 5.561 Å, *c*_BNF_ = 6.832 Å, and *a*_NZF_ = *c*_BNF_ = 8.322 Å, which are remarkably smaller than that of BNF (5.567 Å) and NZF (8.335 Å). The scanning electron microscopy (SEM) micrographs of the top layer of both BNF/NZF and NZF/BNF composite films are shown in Fig. 3, and the insets are the corresponding atomic force microscopy (AFM) micrographs. Figure 3a displays the surface morphology of the top layer of NZF/BNF, whose regular elliptic crystalline grains are homogeneous and connect tightly, while the grains of the NZF layer shown in Fig. 3b are smaller in size and also homogeneous but the size is smaller than that of the BNF layer, which suggests that there would be a smaller nucleation barrier between NZF and BNF nanofilms compared with the Pt/Ti/SiO_2_/Si substrates, thus influences sensitively the multiferroic properties of such composite films, which agrees well with the following analysis of the ferroelectric, dielectric, and ferromagnetic properties.

**Fig. 4:**
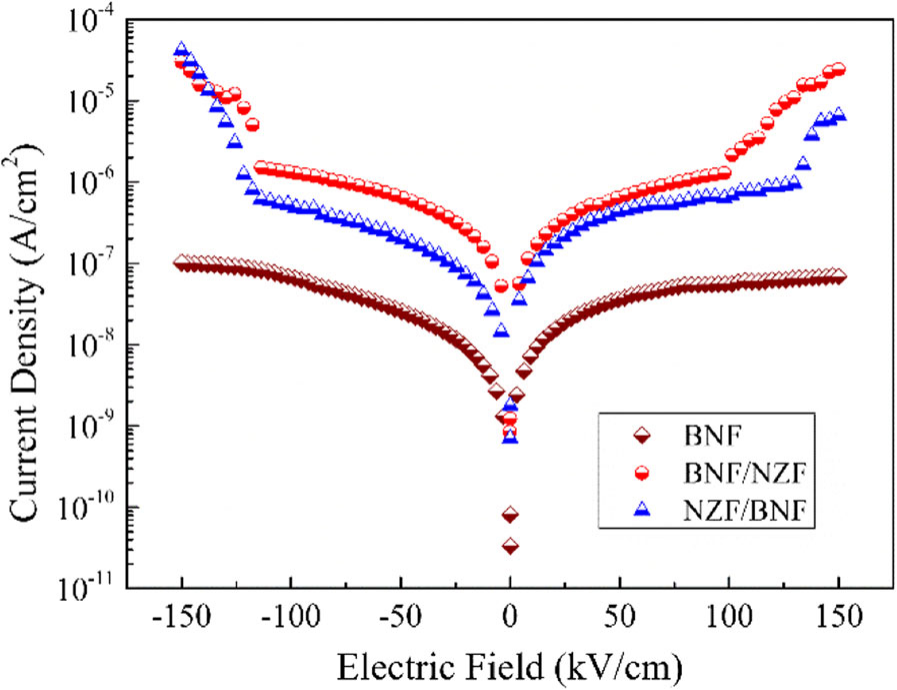
DC leakage characteristics of the BNF monolayer and the bilayer composite films

The DC leakage current characteristics of both BNF/NZF and NZF/BNF composite thin films are given in Fig. 4. It can be seen that the leakage currents of these bilayer magnetoelectric films are significantly higher than that of the BNF monolayer films (1 × 10^−7^ A/cm^2^), and the NZF/BNF composite thin films (3 × 10^−6^ A/cm^2^) have a smaller leakage current density than BNF/NZF (8 × 10^−5^ A/cm^2^), indicating that the insulating property of NZF/BNF is better than that of BNF/NZF because the first layer is critical to the microstructures of the films during the preparation of bilayer nanofilms. In the NZF/BNF structure, the first layer is NZF with low resistance whose grains connect loosely as seen in Fig. 3b, thus affects the insulating property of the later deposited layer, but the leakage current of composite films striking increases when electric field overing is ±100 kV/cm. Conductances of the BNF monolayer in the range of −150~150 kV/cm and bilayer composite films in the range of −100~100 kV/cm depend mainly on electron concentration in transitions from valence band to conduction band generated by thermal activation. The relationship of leakage current and electric field is almost linear, which can be described as [20]

2 $$ J=\delta \cdot E $$

where *δ* is the equivalent conductivity. At the moment, the conducting behavior of films is under the control of the *ohmic* transmission mechanism. But the leakage current of the composite films increases rapidly while electric field overing is ±100 kV/cm, which can be explained by the *Schottky* emission model with deep traps when contact type between electrodes and films is the blocking contact. The *Schottky* barrier at the interfaces will be weakened under an external field, electrons blocked by the *Schottky* barrier are easy to be emitted, and the current density is given by the relation (3) [21]

3 $$ J={A}^{*}\times {T}^2 \exp \left[\frac{-q\left({\varphi}_B-{\beta}_S\sqrt{E}\right)}{KT}\right] $$

where A^*^ is the *Richardson constant* which is relevant to the carrier mobility, *T* is the *Kelvin temperature*, *q* is the effective electron charge, φ_*B*_ is the ideal *Schottky* barrier, *ε*_*0*_ is the permittivity vacuum, *ε*_*i*_ is the relative dielectric constant, and *K* is the *Boltzmann* constant.

**Fig. 5:**
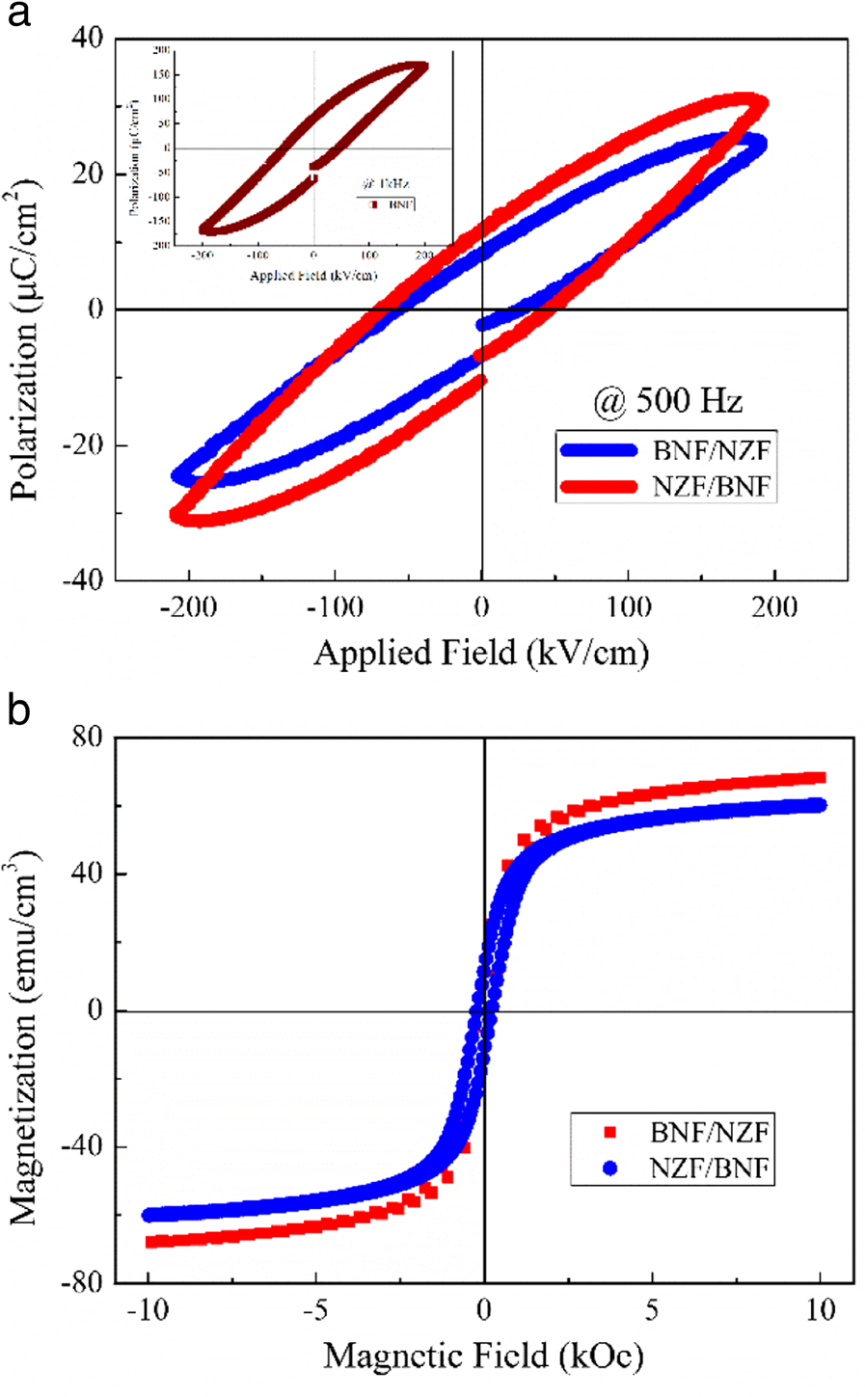
**a** Ferroelectric (P-E) and **b** ferromagnetic hysteresis loops (M-H) of the bilayer composite films. The *inset* in **a** shows the P-E curve of the BNF monolayer tested under 1 kHz

The ferroelectric (P-E) and ferromagnetic hysteresis loops (M-H) of both BNF/NZF and NZF/BNF double-layered films at room temperature are given in Fig. 5, and the inset in Fig. 5a shows the P-E of the BNF monolayer films tested under 1 kHz. As seen from Fig. 5a, the BNF sample shows a well-defined ferroelectric loop than the composite films, the saturated and remnant polarizations (Ps = 169.51, Pr = 61.21, μC/cm^2^) are higher than BNF/NZF (28.33 and 9.62) and NZF/BNF (32.04 and 11.17), but the coercive field (Ec = 44.86 kV/cm) is greater than BNF/NZF (32.09) but less than NZF/BNF (48.72). Obviously, ferroelectricity of the composite films depends mainly on the BNF layer, which originated from the locomotion of Bi^3+^ and Nd^3+^ along the *a* axis of FeO_6_ octahedron [22], the introduction of the NZF layer worsens the overall ferroelectricity, and for the composite films, a higher polarization exhibited in the NZF/BNF films, which is due to the smaller leakage current density caused by a better insulating property [23] of the NZF/BNF films. Antiferromagneticity of the BNF layer is much higher than that of BFO films for the smaller grain size [24, 25], coming from the spinning of Fe^3+^ in reverse order with adjacent ions. As is shown in Fig. 5b, the bilayer composite films exhibit typical magnetic hysteresis loops, as well as magnetizations, thus indicating the presence of an ordered magnetic structure. It is obvious that both BNF/NZF and NZF/BNF structured composite films have the comparable magnetic hysteresis loops, the remnant magnetizations (Mr = 21 emu/cm^3^), and the coercivity (Hc = 67.13 kOe) and only the saturation magnetization (Ms) of BNF/NZF (67.22 emu/cm^3^) is slightly higher than that of the NZF/BNF films (64.83 emu/cm^3^), which indicates that these double-layered composite films have similar magnetic properties.

**Fig. 6:**
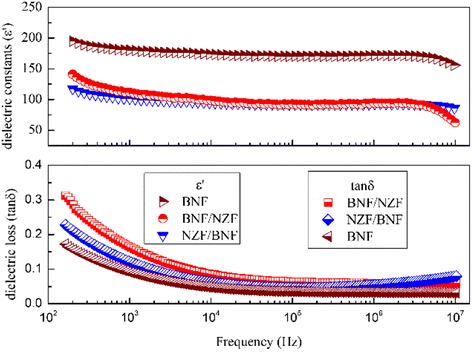
Dielectric spectroscopy of the BNF monolayer and the bilayer composite films

Dielectric spectroscopy of the composite films from 100 to 10^7^ Hz is shown in Fig. 6. The relative dielectric constant (*ε′*) decreases (*ε*_BNT_*′* = 198, *ε*_BNF/NZF_*′* = 149, and *ε*_NZF/BNF_*′* = 124 at 100 Hz), and the dielectric loss (tan*δ*) increases (tan*δ*_BNT_ = 0.16, tan*δ*_BNF/NZF_ = 0.31, and tan*δ*_NZF/BNF_ = 0.23 at 100 Hz) than that of the BNF monolayer film throughout the whole test frequency, and the composite films show a typical frequency-dispersion property [26]. The *ε′* of BNF/NZF is higher than that of NZF/BNF before 10^4^ Hz but significantly less after 3 × 10^6^ Hz and basically the same in this range, and the tan*δ* dives before 10^5^ Hz but then keeps unchanged; only the tan*δ* of NZF/BNF raises a little after 10^6^ Hz. This effect of dielectric constant can be explained by not only the *Seepage* theory but also the *Maxwell-Wagner* (M-W) surface polarization theory. Polarization charges are produced by the asymmetry of the dielectric materials, and the dielectric constant is proportional to the numbers of space polarization charges between the two phases [27],

4 $$ Q=\frac{V{\varepsilon}_0\left({\gamma}_1{\varepsilon}_2-{\gamma}_2{\varepsilon}_1\right)}{\gamma_1{d}_2+{\gamma}_2{d}_1}S $$

where *Q* refers to the numbers of surface charges; *V* is the polarization voltage; *S* is the contact area; and *ε*_*i*_, *γ*_*i*_, and *d*_*i*_ represent, respectively, the dielectric constant, the conductivity, and the thickness of the two phases. For the laminar composite films, the contact area has an important influence on the dielectric constant, and the interpenetrating between the ferroelectric and ferromagnetic phase will form coupled defects for the discrepancy of the lattice parameters; if the first layer is NZF whose grains connect loosely as seen in Fig. 3b, the lattice distortion of the BNF films deposited on such a layer will aggravate leading to a higher *ε′*. The tan*δ* indicates the energy consumed in the process converting electric energy into thermal energy in dielectric media. For the BNF, the tan*δ* is mainly decided by the properties of dielectric media, but the introduction of NZF enlarges obviously the loss, which indicates that NZF can also generate heat interiorly, which cannot be ignored for the composite films.

**Fig. 7:**
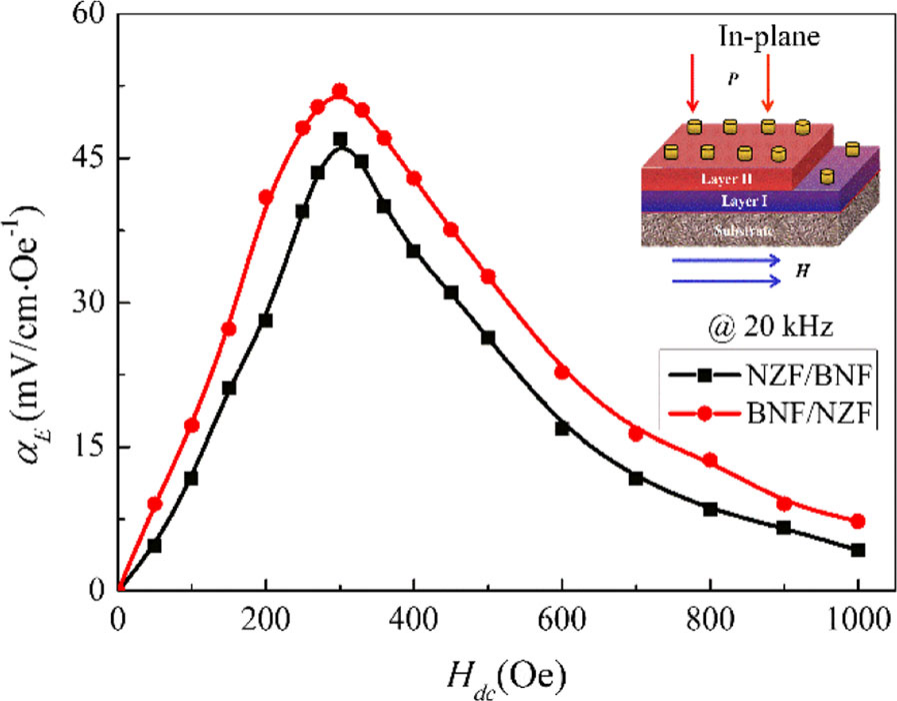
In-plane variation of *α*_*E*_ of the bilayer thin films with *H*_ac_ at magnetic frequency *f* = 20 kHz

The ME coupling of the multiferroic composites mainly arises from the magnetic-mechanical-electric interaction between the magnetostrictive and ferroelectric phases through the stress/strain in the interface [28, 29]. Figure 7 shows the in-plane magnetoelectric coupling of the composite films (the external magnetic field is parallel to the films while the polarization field is perpendicular to the samples) at a fixed ac magnetic frequency *f* = 20 kHz, measured at room temperature. Film samples need to be polarized (to enhance the piezoelectric property of films) and magnetized﻿ at first [19]. The alternating magnetic field *H*_ac_, provided by a long straight helix tube, is to induce the coming about of a ME coupling electric field. As shown in Fig. 7, the magnetoelectric coupling coefficient *α*_*E*_ rockets with the increase of the dc bias magnetic field *H*_dc_ provided by an electromagnet to eliminate the influence of the external magnetic field and the frequency-doubling effect caused by magnetostrictive materials and reaches to the vertex (*α*_BNF/NZF_ = 51.32, *α*_NZF/BNF_ = 47.18, mV/cm·Oe^−1^) at 300 Oe where the effective magnetostrictive strain *λ* approaches its saturation [30]; then, it will produce a nearly constant electric field in the BNF ferroelectric phase, and thereby, *α*_*E*_ descends gently to the bottom with the further increase of *H*_dc_. The value of *α*_*E*_ is much higher than that of the Bi_3.15_Nd_0.85_Ti_3_O_12_-NiFe_2_O_4_ bilayer films [24]. The larger *α*_*E*_ of the BNF/NZF composite film might be due to the enhancing of the interfacial coupling. In the BNF/NZF and NZF/BNF bilayer structures, displacements of atoms at the interface caused by ferroelectric instability alter the overlap between atomic orbitals at the interface, which affects the interface magnetization. This produces a ME effect, the essence of which is the sudden change in the interface magnetization induced by the polarization reversal in the ferroelectric layer under the influence of an applied electric field [31]. For the BNF/NZF films, a possible weak interface coupling between BNF and NZF will decrease the *α*_*E*_ for the NZF/BNF films. On the contrary, for the BNF/NZF films, the later BNF precursor overlays and permeates into the loosen NZF layer, enhanced effectively the interface bonding and resulting in a larger *α*_*E*_.

## Conclusions

In conclusion, bilayer ME nanofilms BNF/NZF and NZF/BNF have been deposited on the Pt(111)/Ti/SiO_2_/Si(100) substrates via sol-gel and a subsequent rapid thermal process. Phase composition, microstructure, and ferroelectric, dielectric, ferromagnetic, and ME coupling properties of the composites have been confirmed at room temperature. The nucleation barrier caused that it is easier for NZF and BNF to grow on each other rather than on the surface of Pt/Ti/SiO_2_/Si. Because the layer deposition sequences have a great influence on the properties of the bilayer films (the interfacial effect), such heterostructures present a little difference on the ferroelectric, ferromagnetic, and dielectric properties, as well as the ME coupling coefficient *α*_*E*_. The BNF/NZF films showed better ferromagnetic, ME coupling properties and dielectric constant but larger leakage current and dielectric loss than the NZF/BNF samples.
